# Scaling up Locally Adapted Clinical Practice Guidelines for Improving Childbirth Care in Tanzania: A Protocol for Programme Theory and Qualitative Methods of the PartoMa Scale-up Study

**DOI:** 10.1080/16549716.2022.2034136

**Published:** 2022-03-21

**Authors:** Jane Brandt Sørensen, Natasha Housseine, Nanna Maaløe, Ib Christian Bygbjerg, Britt Pinkowski Tersbøl, Flemming Konradsen, Brenda Sequeira Dmello, Thomas van Den Akker, Jos van Roosmalen, Sangeeta Mookherji, Eunice Siaity, Haika Osaki, Rashid Saleh Khamis, Monica Lauridsen Kujabi, Thomas Wiswa John, Dan Wolf Meyrowitsch, Columba Mbekenga, Morten Skovdal, Hussein L. Kidanto

**Affiliations:** a Global Health Section, Department of Public Health, University of Copenhagen, Copenhagen, Denmark; b Medical College, Aga Khan University, Medical College East Africa, Dar Es Salaam Campus Tanzania; c Department of Obstetrics and Gynaecology, Hvidovre University Hospital, Hvidovre, Denmark; d Comprehensive Community Based Rehabilitation in Tanzania Tanzania; e Athena Institute is the department, Athena Institute, VU University, Amsterdam, The Netherlands; f Department of Obstetrics and Gynaecology, Leiden University Medical Centre, Leiden, The Netherlands; g Department of Global Health, George Washington University Milken Institute School of Public Health, Washington DC, US; h Medical college, Aga Khan University, School of Nursing and Midwifery East Africa, Dar Es Salaam Campus Tanzania; i Section for Health Services Research, Department of Public Health, University of Copenhagen, Copenhagen, Denmark

**Keywords:** Practice theory, obstetrics, intervention, co-creation, respectful maternity care

## Abstract

Effective, low-cost clinical interventions to improve facility-based care during childbirth are critical to reduce maternal and perinatal mortality and morbidity in low-resource settings. While health interventions for low- and lower-middle-income countries are often developed and implemented top-down, needs and circumstances vary greatly across locations. Our pilot study in Zanzibar improved care through locally co-created intrapartum clinical practice guidelines (CPGs) and associated training (the PartoMa intervention). This intervention was context-tailored with health-care providers in Zanzibar and now scaled up within five maternity units in Dar es Salaam, Tanzania. This PartoMa Scale-up Study thereby provides an opportunity to explore the co-creation process and modification of the intervention in another context and how scale-up might be successfully achieved. The overall protocol is presented in a separate paper. The aim of the present paper is to account for the Scale-up Study’s programme theory and qualitative methodology. We introduce social practice theory and argue for its value within the programme theory and towards qualitative explorations of shifts in clinical practice. The theory recognizes that the practice we aim to strengthen – safe and respectful clinical childbirth care – is not practiced in a vacuum but embedded within a socio-material context and intertwined with other practices. Methodologically, the project draws on ethnographic and participatory methodologies to explore current childbirth care practices. In line with our programme theory, explorations will focus on meanings of childbirth care, material tools and competencies that are being drawn upon, birth attendants’ motivations and relational contexts, as well as other everyday practices of childbirth care. Insights generated from this study will not only elucidate active ingredients that make the PartoMa intervention feasible (or not) but develop the knowledge foundation for scaling-up and replicability of future interventions based on the principles of co-creation and contextualisation.

## Background

Despite considerable evidence on the effectiveness of local, low-cost clinical interventions in improving survival and safety during childbirth, maternal and perinatal mortality and morbidity remain a major public health catastrophe in low- and lower-middle-income countries (LLMICs) [1–3]. Acknowledging that one size does not ‘fit all’, solutions tailored to local circumstances are warranted [4]. While such processes of local co-creation are promising, a key challenge remains: how do we scale-up interventions that have been co-created and proven to work in one setting and then adapt them to another? What levels of additional co-creation and modification are required and necessary to up-scale the intervention feasibly and successfully? The PartoMa intervention provides a unique opportunity to explore these critical questions.

Both within and beyond maternal health, research-based evidence and benefits are difficult to implement in healthcare [5]. Typically, the translation from research to reality focuses on developing rather generic interventions. The misfit of such interventions, however, in settings where they are most needed, results in under- or over-use, with unforeseen side effects [6]. Development of large-scale Clinical Practice Guidelines (CPGs) for LLMICs has been a central international strategy to strengthen clinical practice during pregnancy and childbirth [7]. While these CPGs are typically designed by international, external experts, local representatives, may not be adequately, if at all, involved, and post-implementation evaluations are largely neglected [6]. Though encouraged by international CPG developers, national or subnational adaptations become unrealistically complex and resource intensive, often resulting in guidelines that are too generic and unsuitable for the context [8,9]. Consequently, frontline health care providers are left with guidance incompatible with local realities, heightening the risk of causing unintended harm [6].

Whilst pilot studies from low-resource settings report promising improvements in childbirth care as a result of contextualized and adapted CPGs, only few are up-scaled. There is a need to focus on how effective interventions can be replicated and taken to scale across contexts and settings. A scale-up study of the safe childbirth checklist programme in India emphasizes important accelerators for successful intervention scale-up [10]. These include 1. testing of interventions in real resource-constrained settings in the current health system, social and political context; 2. applying a simple, context appropriate and time-sensitive research approach for evaluation; 3. testing the intervention at substantial scale and in diverse settings; 4. using a contextual adaptive and iterative approach for implementation; and 5. continuously sharing data with key stakeholders, harmonizing them with ongoing activities 8. An important feature in the different components of a complex project, including intervention development, pilot-testing and upscaling, is to actively listen to and involve relevant frontline people. This involves consideration of local realities and experiences on the ground, such as history, governance style, social dynamics, and (dis)trust in health systems, both by frontline health care providers and individuals in need of care [11,12].

The above sketched disconnect persists between how the global health community typically frames problems and the lived experiences of people and health care providers in specific settings [11]. Implementation research from low-resource health systems that takes context into account remains scarce [13]. To close the gaps between externally derived CPGs and low-resource clinical realities, we developed and conducted the PartoMa pilot study in Zanzibar, Tanzania (2014–2018) [14–16]. To ensure that the intervention was tailored to its context and end-users, the guidelines were co-created with local birth attendants. In the co-creation process, significant changes were made to international guidelines in order to render them implementable and context-appropriate. The CPG also underwent external peer-review by specialists in obstetrics, midwifery and neonatology, who had working experience in low-resource maternity units. The final product was thus both evidence- and context-informed and involved re-negotiation of best possible practice. The CPG was then implemented through a low-dose, high-frequency training [17] and showed promising results [14–16]. The intervention developed and implemented during the PartoMa pilot study remains active in Zanzibar. These findings raise the pertinent question: How do we take this intervention approach to scale?

### Study objectives

Following the PartoMa pilot project in Zanzibar, we now commence a scale-up study in five maternity units of Dar es Salaam, Tanzania, with the overall objective to explore if and how the PartoMa intervention can contribute to catalyse safe and respectful childbirth care practices. As further described in the overall protocol [18], this will be achieved through four main steps: I. A mixed-methods situational analysis to explore factors affecting care; II. Developing co-created contextual modifications to the PartoMa CPG and training from Zanzibar, based on findings from step I; III. Implementation and evaluation of the modified PartoMa intervention; and IV. Development of a framework with relevance to multiple contexts, for co-creation/co-modification of context-specific CPG and training, within and beyond maternal health [18].

To elucidate pathways to impact, this protocol paper presents our programme theory including the theoretical frame of *practice theory* to provide an explanation for the mechanisms of change, and qualitative study design to explore contextual factors affecting the intervention co-creation and implementation. Specifically, we set out to explore practices and context of childbirth care and what are acceptable practices to both birth attendants and women giving birth. Thus, we conceptualise contextual factors as spheres of influence in the co-creation of intervention adaptations and in the implementation process. This allows Us to explore how these spheres work together, and how they intersect to alter clinical and social practices. We hereby adopt a practice perspective to account for both our programme theory and to qualitatively investigate and explain possible shifts in clinical practice for improved childbirth care.

The specific aims of the PartoMa Scale-up Study presented in this protocol are

- To develop a comprehensive programme theory outlining the hypothesised pathways to change, based on contemporary theories of social practice
- To examine pre-intervention clinical practices of childbirth care from the perspectives and experiences of women who have recently given birth, birth attendants and hospital managers, including an analysis of how the COVID-19 pandemic has influenced birth attendants’ ability to provide care [Step I, situational analysis]
- To explore how the PartoMa Scale-up Study is contextualized using participatory and co-creative processes [Step II, co-creation]
- To investigate how the processes of context-modifying and availing physical copies of birth guidelines and training induce shifts in safe and respectful childbirth care [Step III, implementation and evaluation]
- To draw on research findings to develop a practice-based approach for co-creation and implementation of CPGs and associated training that may be of relevance within and beyond maternal health [Step IV, framework]

## Methods and Analysis

### Research group

Organization of the overall PartoMa Project study team is described in the main study protocol [18]. Suffice to note that the team consists of an interdisciplinary group of scientists and clinicians, consisting of medical doctors, epidemiologists, statisticians, midwives and social scientists, the majority of whom are familiar with the Tanzanian context. The team is based on collaboration between Comprehensive Community-Based Rehabilitation in Tanzania (CCBRT), a non-governmental organization (NGO), and academic institutions in Tanzania, the Netherlands and Denmark.

### Study setting and context

The PartoMa Project will be implemented in five government-run hospitals in Dar es Salaam, the largest and rapidly growing city of Tanzania. They include regional referral hospitals (Temeke, Amana and Mwananyamala) and two district hospitals (Mbagala Rangi Tatu and Sinza). Two of these hospitals will be selected purposively and followed ethnographically throughout the study. Birth attendants and women who have recently given birth in one of the five sites will be included in the co-creation part (step II) to capture contextual elements across hospital sites, including leadership, and the historical, geographical, economic, cultural and spiritual fabric.

The hospitals are typical examples of overburdened urban maternity units in a lower-middle-income country, where the quality of care is impacted by lack of physical infrastructure, human resources and supplies. In 2019, the selected hospitals jointly cared for 40% of all hospital-based births in Dar es Salaam [19]. Each birth attendant typically takes care of multiple women simultaneously. Being public hospitals, they primarily serve women of lower socioeconomic backgrounds (17).

Several efforts have been made to improve maternal and perinatal health in hospitals in Dar es Salaam. A public–private partnership between regional health authorities and CCBRT saw that positive changes in quality of care were related to further strengthening of health care providers’ competencies, stable access to equipment and medicine, improved data quality and decongestion of overburdened facilities. While these efforts resulted in significant improvements in the 22 health facilities participating in the initiative [19], an in-depth exploration of the mechanisms of change would be useful. Although qualitative research from the study sites is scarce, other studies focusing on Tanzanian women’s experiences of giving birth and birth attendants’ clinical practices, work conditions and perceived challenges have found examples of both respectful and disrespectful care [20,21]. These practices were mostly related to health system challenges, including difficult physical environment, lack of supportive supervision and supplies and lack of motivation among health care providers [20,22]. Furthermore, high patient flows and resource shortage are likely drivers of the dangerous coexistence of ‘too little, too late’ or and ‘too much, too soon’. These conditions underpin suboptimal care with unachievable workloads, lack of routine surveillance and underuse of the partograph as a decision-making tool during labour and doubtful caesarean section indications [23]. Birth attendants explained how suboptimal practices were outside of their control and responsibility, but caused by dysfunctional team work, insufficient support from senior colleagues and fear of being blamed by seniors and management in case of poor outcome [24,25]. As in other sub-Saharan African countries, birth attendants in Tanzania demand access to professional development, staff support and supervision, for which context-tailored clinical guidance is central [26]. Notably, in this already resource-constrained context, it is likely that COVID-19 has added to the burden on birth attendants [27]. On top of providing maternity care, birth attendants have to detect and manage COVID‐19 infection and prevent its spread with even fewer resources [28]. Overall, this leads to our initial hypothesis of how routinized socio-material practices drive suboptimal care.

### Programme theory and conceptual framework

**Figure 1.**
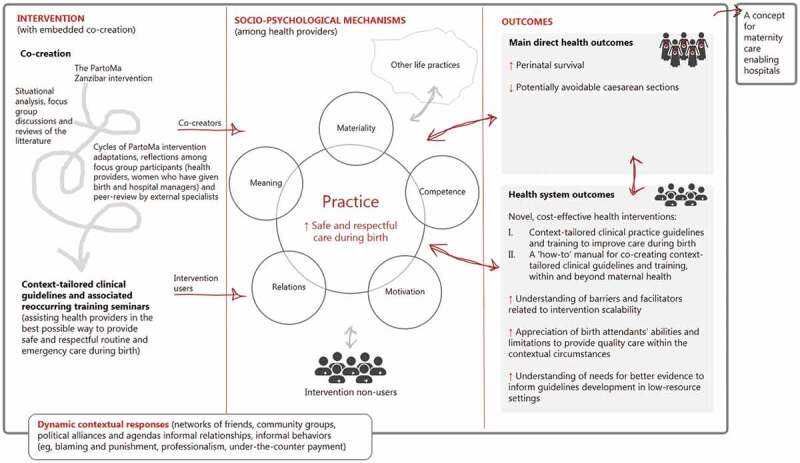
The PartoMa intervention’s programme theory. It is hypothesized that the intervention, with embedded co-creation, improves clinical practice and the desired health and health system outcomes through a reconfiguration of interacting mediators, which are divided into practice theory’s five analytical domains: Meaning, Materiality, Competence, Motivation, Relations as well as other life practices. These domains are further explained in Figure.

The PartoMa intervention has all the features of a non-linear complex intervention: it has multiple interacting components and actors, is influenced by context (including COVID-19 related changes) in all phases, seeks adaption to context, contains feedback loops and relates to multiple outcomes – as illustrated in Figure 1. Following the recommendations for developing and conducting complex intervention and implementation studies, the study team developed a programme theory, hypothesising how the intervention might instigate change, including the contextual conditions required for the changes to come about [5,29]. The programme theory is based on experiences from the PartoMa pilot study conducted in Zanzibar [14], existing context-relevant literature, informal stakeholder conversations and workshops with project team members, of which some are familiar with the study sites in Dar es Salaam.

In exploring the initial hypothesis of routinized socio-material practices driving suboptimal care, we draw on contemporary theories of social practice. Common to such theories is that they treat practices as primary units of enquiry and provide conceptual tools and language to explore and understand the actions of people from a social and structural perspective. In practice theory, it is argued that understanding how human behaviours – such as those surrounding childbirth care – take hold, is a matter of ‘understanding how the many practices that are reproduced in the course of daily life are synchronised and coordinated, and how some become more deeply embedded than others’ [30]. Practice theory thus helps us unpack everyday practices and experiences that influence birth attendants’ (dis)engagement with respectful and safe childbirth care (including current COVID-19-related practice and experience), across socio-ecological spheres of influence, space and time [31].

**Figure 2.**
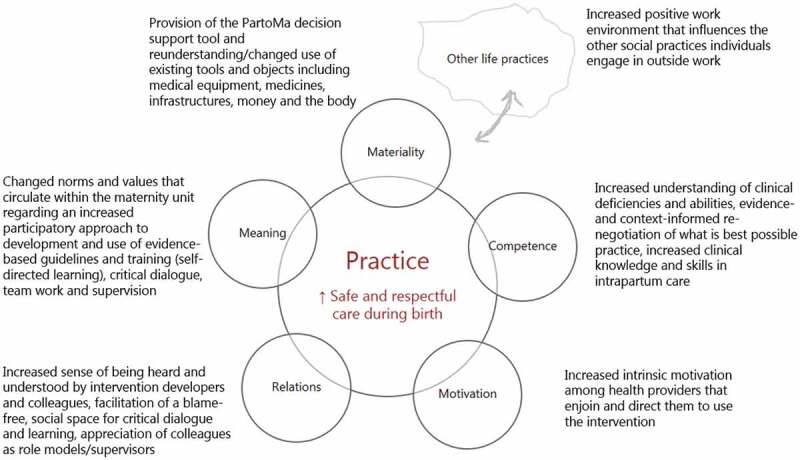
Overview of the expected socio-psychological mechanisms the intervention will facilitate using elements of practice theory.

For this study, we draw on the social practice framework developed by Skovdal to support practice-based programme theory and research for health interventions in low-resource settings [32]. Drawing on the works of contemporary social practice theorists, Skovdal presents a two-step ‘table of questioning’ for interrogating and understanding the intersecting spheres of influence (step 1) and other everyday practices (step 2) that shape health care [30]. We draw on this framework to explore how the absence, presence or introduction of contextual factors meanings, materials, competencies, motivation and relations (social practice resources that we introduce below and in Figure 2)

and other everyday practices come together to affect childbirth care. The latter allows us to recognise that birth attendants participate in a number of interwoven practices that may obstruct or support safe and respectful childbirth care [33]. We hypothesise that by involving birth attendants and stakeholders in a participatory process of modifying the PartoMa CPG (referred as the co-creation process), we are more likely to shift or reorganize the type of contextual factors, or spheres of influence, that routinize safe and respectful care at birth. Here, we elaborate on the social practice resources and other everyday practices – as far as they relate to childbirth care – that we hypothesise the intervention might affect.

### Meanings

Meanings refer to norms, values, communitarian convictions, social representations and ideologies that circulate within the maternity units. The PartoMa intervention seeks to facilitate change in meanings. This starts with the co-creation process, which we expect will bring transparency to what is expected of a birth attendant and facilitation of social spaces for critical dialogue, which may contribute to re-negotiations of what constitutes best possible intrapartum care within the existing resource-constrained health care system. The co-creation process is also likely to facilitate ownership of both problems and solutions, allowing participants to rethink norms and values around their roles and responsibilities.

### Materials

Materials include ‘things’ such as tools, objects and infrastructure that make up the maternity units and the resources available. We hypothesise that the PartoMa intervention will contribute to a number of shifts in the availability of ‘materials’. Specifically, the intervention will provide birth attendants with a physical decision-support tool: the updated and co-adapted version of the PartoMa pocket book (Publichealth.ku.dk/partoma). This book constitutes material technology and is aimed to be locally achievable, practical, relevant, unambiguous, brief and easy to understand. Birth attendants in the PartoMa pilot study in Zanzibar referred to the booklet as ‘a friend in the pocket’ (unpublished data). We also believe that the co-creation process and intervention will bring about new ways of utilising existing tools and objects, including medical equipment, other CPGs, medicines, infrastructure, money and the body. The partograph has been available for many years to monitor vital signs and progress during birth. While the partograph is often exclusively seen in clinical practice as a medical record (22), the PartoMa intervention emphasizes its use as an integrated, early at a glance warning tool for the well-being of women, their babies and labour progress.

### Competencies

Competence refers to the different understandings of childbirth care practices, as well as practical expertise, know-how and skills that birth attendants and hospitals need to perform. In addition to obtaining access to much needed resources (‘materials’), it is vital that birth attendants are aware of how to use them effectively. The PartoMa intervention aims to provide a positive, welcoming, social, safe, and enabling environment for participants to share and process information, taking ownership of previously alien or decontextualised medical information. Participants in the seminars in the PartoMa pilot study in Zanzibar referred to the training sessions as ‘therapeutic afternoons’ (unpublished data). Active participation with critical reflection was encouraged during the Zanzibar seminars, for instance regarding how to provide best possible triage as each birth attendant cares for multiple labouring women simultaneously. The PartoMa intervention integrates common routine and emergency care as well as guidance for how to prioritize, which in Zanzibar increased clinical knowledge, competencies, and skills in decision-making during intrapartum care [14].

### Motivation

Motivation refers to desires and visions that drive and direct people and organisations to engage with a practice. We hypothesise that co-creation as well as the participatory format of the training seminars will lead to increased intrinsic ownership and motivation to practice childbirth care in a different way [34]. Further, we hope that participants will invest themselves in the process and thereby take ownership. The participatory processes and the intervention itself may facilitate ‘bottom-up’ insights and formulations of positive social actions to improve childbirth care, and foster solidarity and the motivation needed to act, care and support shifts in the practice of childbirth care. In Tanzania, birth attendants’ motivation to engage in performance enhancing activities has been linked to financial gains [35]. A remarkable finding from the PartoMa pilot study in Zanzibar, however, was that ‘low-dose, high-frequency training’ which took place outside working hours, without per diems or allowances, still had a consistently high attendance rate (an average of 60–70% of staff) [14]. It thus appeared that birth attendants chose to participate in seminars not because it provided them with extrinsic material rewards, but because it created immaterial intrinsic rewards. Active participation of birth attendants in the training seminars is further expected to have a positive influence on their commitment and job-satisfaction [36]. Related to adult learning theory, it may even be argued that attending during their free time without allowances, amplified the birth attendants’ experience of self-directed learning, meaning that their gain in knowledge and skills was largely within their control and a free choice, thereby enhancing their striving and acceptance of a personal responsibility for own learning [37]. We hypothesise that this ‘new’ enabling environment with increased intrinsic motivation will also affect birth attendants who do not participate in the co-creation process as well as non-users of the intervention.

### Relations

Relations refer to the quality of relationships, partnerships, formal and informal networks, and group dynamics that characterise the maternity units. We hypothesize that the intervention will bring a change in social relations – both among birth attendants and the relationship they have with the women giving birth. A crucial assumption of the PartoMa intervention is that the social practice of interdisciplinary teamwork is key to obtaining changes in care, considering constraints in human resources. Through the participatory approach, birth attendants may experience an increased sense of being heard and understood. The blame-free, social space for critical dialogue and learning is expected to bring appreciation of colleagues doing well as role models and supervisors.

### Other life practices

Birth attendants participate in a number of interwoven life practices that may obstruct or support their clinical practices, e.g. many of the primarily female birth attendants have profound family obligations or additional employment in the private sector, which is likely to influence their work in public hospitals [38]. Having to stay after work for the PartoMa seminars might have a negative influence on family obligations and finances. On the other hand, we expect that an enabling working environment will positively influence other social practices in, during- and outside clinical practice.

### Qualitative components of the four-step study design

The programme theory led to developing the four-step study design (Table 1). Together, these investigations will help build a comprehensive understanding of the development and implementation process and impact as well as scalability and replicability analyses of the PartoMa Scale-up Study. While the overall study design for each step is presented in the general protocol [39], we here focus in-depth on the qualitative components.

**Table 1.** Overview of the four steps of the PartoMa scale-up study (I. situational analysis, II. Co-Creation, III. Intervention, and IV. Development of a framework based on findings) and the qualitative components associated with each phase

Phase	What	Why	How	Where
I. Situational analysis	Understand how women, birth attendants and hospital managers experience, interpret and engage with current birth care and postpartum clinical care.	To explore the role of people, structures and materialities in shaping the patterns of daily hospital life that affect how intra- and postpartum care is clinically practiced before the intervention.	Through in-depth interviews, FGDs and observations.	Two hospitals in Dar es Salaam.
II. Intervention co-creation	(A) Develop context-modifications for the PartoMa intervention of CPGs and training to reflect birth attendants and labouring women’s needs and circumstances. (B) Understand the components of the intervention that survive between settings; explore resources and experiences hospital stakeholders bring to the process; and how co-creators experience the co-creation process.	(A) To make the PartoMa intervention relevant to the Dar es Salaam hospitals setting. (B) To capture and understand the process of co-creating the PartoMa guidelines to fit the local realities.	(A) Through iterative cycles of focus group evaluation, intervention modifications and external peer review. (B) Through an ethnographic approach, including observations and FGDs.	The five selected hospitals in Dar es Salaam, making one adapted PartoMa intervention.
III. Intervention implementation and evaluation	Understand opportunities and challenges in the implementation and scale up of the PartoMa intervention.	To unpack pathways and contextual factors affecting engagement with the PartoMa guidelines.	Through in-depth interviews and observations.	Two hospitals in Dar es Salaam.
IV. Development of framework	Zoom out and utilize the findings of the process and implementation of PartoMa in order to make them attainable for other settings.	To develop a framework for co-creating and implementing CPGs and associated training.	By utilizing the findings from step I, II and III.	The five selected hospitals in Dar es Salaam.

## Situation analysis

We will first conduct a review of context-relevant literature, anticipating that only few qualitative studies have focus on the hospitals and practices under our investigation. The review will be followed by a thorough situational analysis among women who have recently given birth, birth attendants and hospital managers, as well as mapping of relevant stakeholders. Here, qualitative methodology will be utilized to explore the situation on the ground by zooming in on birth attendants’ everyday routines, work motivation and how they interpret and engage with current clinical practice during birth. Specifically, focus will be on absence and presence of factors that shape childbirth care as well as opportunities for improvement of provided care. The analysis will also include an investigation of the impact of COVID‐19 on routine clinical practices in the maternity wards.

## Co-creation process

The PartoMa CPG booklet from the Zanzibar pilot study will be shared with co-creators. Building on the Zanzibari version, a first draft of the PartoMa Dar es Salaam CPGs will be developed by co-creators and the research team, incorporating findings from step I. It should be noted that birth attendants are co-creators, implementers and users of the intervention – and some of them part of the research team – which is all part of making the intervention complex. Iterative cycles will take place, consisting of focus group discussions (FGDs) where co-creators share their perspectives on the booklet to inform further modifications, which will then be reviewed by an external expert panel, before being returned to the co-creators for further input. Focus of the PartoMa Scale-up Study is to examine this co-creation process, including clinical care elements that ‘survive’ or do not survive between settings. We will also explore how experiences of stakeholders inform modifications, as well as how the co-creators experience the process itself. The co-creation process is thus both vital in developing the intervention as well as an instrumental part of the actual intervention. The co-creation process will be ethnographically explored, with a researcher participating in, and observing, the co-creation activities.

## Intervention implementation and evaluation

The aim is to explore how co-creation and implementation of the PartoMa CPGs affect team work and collaboration as well as individual birth attendants’ participation, motivation and practices and thus unpack the pathways and contextual factors contributing to potential measurable outcomes of the PartoMa Scale-up Study. By engaging birth attendants, hospital managers and administrators with different qualitative methods, absence, presence and introduction of factors, such as implementation of the PartoMa intervention and other life practices shaping childbirth care, will be explored. Collective experiences and perspectives on strengths and limitations of the PartoMa CPGs and associated activities will be explored. Following qualitative analysis we will be able to determine whether and how the co-creation process has contributed to adaptability, acceptability, usability, scalability and transferability of the PartoMa CPGs.

## Development of framework

Based on both qualitative and quantitative findings from steps I, II and III [18], and findings from the PartoMa pilot study in Zanzibar, opportunities and barriers in co-creating, implementing and upscaling CPGs and training will be analysed. Thereby, the aim is to develop a framework for co-creating CPGs and associated implementation strategies, within and beyond maternal health.

### Data collection

Practice theory directs attention towards methodological tools that facilitate in-depth exploration and observations of what actually happens in the performance of practices [40]. Thus, informed by our programme theory, we draw on a range of qualitative methods relevant to explore the outlined steps of the study.

### Participant observations

To understand clinical practices and leadership culture, participant observations will be conducted to gain an insider-understanding of social and clinical practices and sensitive topics that might be difficult for participants to discuss in interviews [41]. Observations will be carried out during I. Situational analysis, II. Co-creation process and III. Intervention implementation and evaluation to explore shifts in practices.

For steps I–III, participatory observations will be carried out by asking permission to ‘shadow’ representatives of hospital management, birth attendants and women in the maternity wards in the selected hospitals. Informal interviews will be conducted with a focus on both birth attendants’ and managers’ tasks in the hospital, their perceptions of the PartoMa intervention and understanding of opportunities, needs and barriers to perform optimal intrapartum care. To gain insight on women’s experiences of care, pregnant women presenting to the maternity ward for delivery will be observed from the time they arrive to the ward up to when they are released after delivery. The aim is that observations and dialogues will happen in the natural environment and processes of every-day management. This promotes relaxed and routine-like conversations, with less disruption. Observations and informal conversations will be conducted by a medical doctor and a social scientist, thus ensuring a holistic view on the explored practices. Number of participants included for this component will be approximately 20 birth attendants and 5 managers, though recruitment will continue until data saturation is reached.

Observations will also be conducted during the co-creation process and during the PartoMa seminars, with a view to unpack the various practices that shape intrapartum care and engagement with the PartoMa intervention.

### Exploratory semi-structured interviews

Exploratory semi-structured interviews are relevant when seeking to explore participants’ feelings and beliefs about certain topics. Interviews will be carried out with birth attendants in two of the five study hospitals before (step I) and after intervention implementation (Step III). Birth attendants’ experiences and perspectives of care during birth will be explored, paying particular attention to perceptions of factors influencing the decision-making process on key clinical complications during birth. This will include exploring their knowledge-seeking behaviour, how teamwork is experienced, and birth attendants’ own understanding of needs and barriers to perform intrapartum care. There will be a focus on both intervention components that are perceived not to be working and opportunities for improvement. Furthermore, birth attendants will be interviewed about their experiences and challenges in medical note keeping.

Interviews will primarily be conducted by researchers fluent in Swahili. Approximately five birth attendants will be included for this component, though recruitment will continue until data saturation has been reached. Interviews will take place within hospitals, in a room where privacy can be ensured, and digitally recorded if permitted by participants.

In-depth interviews will also be conducted with women who have given birth in one study hospital to explore their experiences and perceptions of giving birth in the hospital setting. Interviews will be conducted by researchers fluent in Swahili in the facility at least one day after women have given birth. Women who are recovering well and show interest in participating will be identified in the post-natal ward of the hospital. Interviews will be conducted in a location within the hospital that is suitable for ensuring participant’s privacy. Number of participants for this component will be around 10, though recruitment will continue until data saturation is reached.

### Focus group discussions

To investigate collective behaviour and social norms related to practices in the wards, including intrapartum decision-making, FGDs will be conducted with birth attendants during the co-creation process (Step II). Aim is to explore practices of birth attendants in the labour rooms and to co-create and modify the PartoMa CPG and seminars to fit the given context (n = 8). For each iterative cycle of co-creation, FGDs will be held with purposely sampled birth attendants (junior and senior doctors and nurse-midwives) in each of the five hospitals. During each of these FGDs, the updated version of the PartoMa CPG pocket booklet and a video clip of the PartoMa seminars in Zanzibar will be presented to spur conversation. A FGD will also be conducted with women who have recently given birth in one of the two selected hospitals. All FGDs will be moderated and led by two researchers fluent in Swahili and there will be about 6–10 participants in each group.

### Sample and recruitment

One district and one referral hospital and participants will be recruited for steps I and III based on convenience sampling, taking location and time of transport into consideration. Acknowledging hierarchical structure in the hospitals, birth attendants will be selected according to a varied sample in terms of their experience, position and gender. Women will be recruited after birth and before hospital discharge and invited to participate in the interviews.

### Data collection tools

For all in-depth interviews with birth attendants, hospital managers and women who have recently given birth, topic guides have been developed to direct interviews. An FGD guide has been developed to direct discussions. Observational guides have been developed to guide the researchers in observing practices, actions and context. Finally, a topic guide has been produced to explore preferences and input to various aspects of the intervention CPG and seminars to inform areas for adaptation. All topic guides will be pilot tested with similar informant groups and revised accordingly. When possible and with participant consent, interviews and FGDs will be audio-recorded and transcribed into English.

### Analysis

Data will consist of transcripts, field and observation notes that will be uploaded for analysis in NVivo12 [42]. Analysis starts immediately when initiating data collection and continues until the phenomena under study are well understood. An analytical coding framework will be developed, based on inductive coding of randomly selected transcripts as well as deductive themes based on current literature. Thus initially, analysis will be informed by our conceptual framework; however, data and emerging themes will guide which theoretical and empirical directions will be prioritised. Analysis will follow the principles of systematic thematic network analysis where data will be coded to identify themes, ideas and patterns and these codes will be grouped into broader themes and thematic networks [43]. Differences, similarities and contradictions will be explored across settings, actors and type of data collection tools. Specifically, using the approach of thematic network analysis, the researchers will follow these steps: (i) getting familiar with the data; (ii) developing a coding framework; (iii) begin to code data; (iv) finalizing list of codes by merging, splitting, and renaming codes; (v) identifying secondary and (vi) tertiary themes; (vii) illustrating the thematic network; (viii) describing the themes; (ix) verifying and refining the thematic network; and (x) using the thematic network to interrogate and analyse data 34. The analysis will result in several peer-reviewed papers, including mixed-methods as well as an analysis of all findings in combination with clearly articulated potential for up-scale of the PartoMa intervention.

## Ethics and dissemination

Ethical approval is obtained from the Tanzanian National Institute of Medical Research (NIMR/HQ/R.8a/Vol. IX/3324, NIMR/HQ/R.8 c/Vol. I/1679, NIMR/HQ/R.8 c/Vol. I/926). A data management agreement has been signed by the partners involved in storing and analysing data. The study is registered in clinicaltrials.gov (NCT04685668). Research permit is obtained from the Tanzanian commission of science and technology (COSTECH). Other permissions are obtained from the regional medical officer and the participating maternity units.

The aim is to not take birth attendants and students away from critical clinical work to participate in interviews, intervention development, training and other research activities. Thus, interviews will be kept short and in times convenient for participants. Co-creation FGDs will be planned in advance with in-charges at the labour wards, to ensure a minimum of disruption. During implementation, trainings will be repeated multiple days to allow more opportunity for participation. Informed consent will be sought from individuals participating in the study. Further, interviews will be conducted in a non-judgemental manner in a safe environment. For the interviews with women who have recently given birth, special attention will be given to ensure they are comfortable taking part in the study. They will be reminded that they can withdraw from the study at any time. All data will be anonymous, as codes will replace participant names and initials. All researchers will be trained to ensure they adhere to Tanzania Ministry of Health’s guidance on infection prevention control measures, such as wearing of face masks, hand‐hygiene and physical distancing, and to provide education to research participants on COVID‐19 prevention measures.

In addition to publication in peer-reviewed open access journals, dissemination seminars are planned to be conducted in Tanzania and all milestones and main findings will be shared with both Tanzanian and international stakeholders, as well as disseminated at the study website (publichealth.ku.dk/partoma/), linking to Aga Khan University and shared through popular media when possible.

## Conclusion

This study design paper reports on the programme theory and qualitative components of the PartoMa Scale-up Study. Drawing on contemporary theories of social practice, we present a comprehensive programme theory outlining the hypothesised pathways to change. The programme theory will be the foundation for evaluating co-creation and adaptation of the PartoMa intervention as well as the range of effects of it. Furthermore, the programme theory guides us in how to focus the qualitative and quantitative work during each of the implementation steps of the PartoMa Scale-up Study. This involves a focus on women who have recently given birth, birth attendants and hospital management’s meanings, materials, competencies, motivation, relations and other life practices that influence childbirth care. Specific attention is paid to identify elements of co-creation and the intervention, along with context, that most directly affect scalability and transferability of the PartoMa approach. Such exploration of a co-created health intervention and its scale-up is warranted to improve childbirth care in resource-constrained settings.

## Paper context

Context-appropriate, co-created clinical practice guidelines to ensure safe and respectful care during birth in low resource settings are crucial. The PartoMa Project aims to scale up co-created clinical practice guidelines developed in the pilot study phase. The project responds to the need for models of how to facilitate and achieve contextualised, co-created clinical practice guidelines at scale. This protocol presents the programme theory and qualitative methods applied to the PartoMa Project.
